# Physicochemical characterisation of combustion particles from vehicle exhaust and residential wood smoke

**DOI:** 10.1186/1743-8977-3-1

**Published:** 2006-01-03

**Authors:** Anette Kocbach, Yanjun Li, Karl E Yttri, Flemming R Cassee, Per E Schwarze, Ellen Namork

**Affiliations:** 1 Division of Environmental Medicine, Norwegian Institute of Public Health, P.O. Box 4404, N-0403 Oslo, Norway; 2 Department of Physics, University of Oslo, Norway; 3 Department for Chemical Analysis, Norwegian Institute for Air Research, Kjeller, Norway; 4 Center of Environmental Health Research, National Institute for Public Health and the Environment (RIVM), Bilthoven, the Netherlands

## Abstract

**Background:**

Exposure to ambient particulate matter has been associated with a number of adverse health effects. Particle characteristics such as size, surface area and chemistry seem to influence the negative effects of particles. In this study, combustion particles from vehicle exhaust and wood smoke, currently used in biological experiments, were analysed with respect to microstructure and chemistry.

**Methods:**

Vehicle exhaust particles were collected in a road tunnel during two seasons, with and without use of studded tires, whereas wood smoke was collected from a stove with single-stage combustion. Additionally, a reference diesel sample (SRM 2975) was analysed. The samples were characterised using transmission electron microscopy techniques (TEM/HRTEM, EELS and SAED). Furthermore, the elemental and organic carbon fractions were quantified using thermal optical transmission analysis and the content of selected PAHs was determined by gas chromatography-mass spectrometry.

**Results:**

Carbon aggregates, consisting of tens to thousands of spherical primary particles, were the only combustion particles identified in all samples using TEM. The tunnel samples also contained mineral particles originating from road abrasion. The geometric diameters of primary carbon particles from vehicle exhaust were found to be significantly smaller (24 ± 6 nm) than for wood smoke (31 ± 7 nm). Furthermore, HRTEM showed that primary particles from both sources exhibited a turbostratic microstructure, consisting of concentric carbon layers surrounding several nuclei in vehicle exhaust or a single nucleus in wood smoke. However, no differences were detected in the graphitic character of primary particles from the two sources using SAED and EELS. The total PAH content was higher for combustion particles from wood smoke as compared to vehicle exhaust, whereas no source difference was found for the ratio of organic to total carbon.

**Conclusion:**

Combustion particles from vehicle exhaust and residential wood smoke differ in primary particle diameter, microstructure, and PAH content. Furthermore, the analysed samples seem suitable for assessing the influence of physicochemical characteristics of particles on biological responses.

## Background

Exposure to ambient particulate matter has been associated with a number of adverse health effects, including cardiopulmonary morbidity and mortality [1-3] and lung cancer mortality [2]. The mechanisms underlying the observed adverse effects and the responsible particle properties are still not well understood. Application of chemical and physical characterisation of particles in epidemiological studies have, however, linked particle properties like elemental and organic carbon content [4,5] and surface area to mass [6], to negative health effects. Similarly, experimental studies have investigated the influence of particle characteristics on biological responses, and the inflammatory potential of several particle types has been found to increase with the particle surface area [7-9]. Recent reviews of the adverse effects of diesel exhaust particles associate both the carbon particle core and the adsorbed organic compounds with the observed immune responses [10,11]. Studies of inflammatory potential and oxidative stress, on the other hand, indicate that the organic component (content of OC or PAHs) is the responsible particle property [12-14]. Furthermore, studies of the mutagenic potential of urban particulate matter support the biologic plausibility of an association between particulate matter and lung cancer, and that a considerable portion of the mutagenic potential of urban particles is accounted for by PAHs [15,16].

Combustion particles, generated during incomplete combustion of fossil fuels and biomass, consist of tens to thousands of primary carbon particles (diameters ≈ 20–50 nm), leading to a large surface area per mass. The primary particles of carbon aggregates may exhibit an internal turbostratic microstructure, consisting of a concentric arrangement of layer planes with a two-dimensional graphitic crystal structure, lacking the ordered stacking of graphite [17]. High resolution transmission electron microscopy allows for visualization of the 002 lattice fringes of the graphitic layer planes [18]. Turbostratic microstructure has been observed in the model particles carbon black and in particles from diesel exhaust and biomass combustion [17,19-22]. The microstructure of primary particles may give information of the processes involved in particle formation [19], and the particles ability to accumulate potentially reactive substances [23].

In Norway vehicle exhaust and residential wood smoke are the primary sources of particulate air pollution, accounting for more than 65% of the total emissions [24]. Detailed knowledge about particle size, morphology and chemical composition is important to understand the mechanisms by which particles affect human health. Particulate matter samples of vehicle exhaust and wood smoke for use in biological experiments were, therefore, collected. The objective of the study was to characterise and compare combustion particles from these two sources with respect to physical and chemical properties that may influence the biological potency of the particles. Analytical transmission electron microscopy techniques were used to analyse the geometric diameter and microstructure of primary carbon particles. The content of elemental and organic carbon was determined using thermal optical transmittance, and the samples were analysed for selected PAHs by gas chromatography with mass spectrometry (GC-MS).

## Methods

### Sample collection

Vehicle exhaust particles were collected in a motorway tunnel (Oslo, Norway) with a traffic load of approximately 40.000 vehicles/24 hrs. For the particle collection, twelve aerosol monitors (Millipore, MAWP 037 A0), loaded with polycarbonate filters with pore size 0.8 μm and diameter 37 mm, were connected to a pump through a specially designed manifold, resulting in an airflow of 6 l/min through each monitor. The manifold was positioned two meters above the road level, and four meters from the passing traffic. In order to obtain sufficient quantities for the biological experiments, particles were sampled continuously for two weeks, scraped off the filters, and pooled. Samples were collected in two seasons, with and without contributions from cars using studded tires, and designated Tunnel St+ (April 2004) and St- (September 2004), respectively. Both samples contained contributions from cars, trucks and busses fuelled with diesel or gasoline.

A common Norwegian wood stove with single-stage combustion (Jøtul 3, Jøtul, Norway), placed in a laboratory at the Norwegian University of Science and Technology (NTNU, Trondheim, Norway), was used for the collection of wood smoke particles. Approximately 80% of the wood stoves in Norway are old stoves with similar combustion technology, and account for the majority of the particulate emissions from wood combustion. The wood smoke was cooled down by dilution with unfiltered air in a dilution tunnel, according to the Norwegian standard for testing of wood stoves [25]. Wood smoke particles were collected by isokinetic sampling (according to NS3058) on polycarbonate filters with pore size 0.8 μm and diameter 102 mm (Millipore), with an airflow through the filter of 13.3 l/min. Prior to particle collection, the stove was heated for 30 min. Particles were sampled only during high-temperature combustion, obtained by a medium air supply and reloading of the oven approximately every 20 – 30 min. The pump was turned off during the loading process, and turned on when the initially visible smoke from the pipe had ceased. To investigate a possible contribution from particles in the laboratory air to the wood smoke sample, the concentration of particulate matter in the laboratory was measured using a Respicon^® ^virtual impactor (TSI Incorporated, USA), and was found to be 69 μg/m^3^. This contribution was calculated to be smaller than 0.005%, and considered negligible. Wood smoke particles were scraped off a total of 25 filters, pooled and designated Wood.

Diesel exhaust particles (SRM 2975, Industrial Forklift), were purchased from the National Institute of Standards and Technology (Gaithersburg, MD), and designated Diesel. Since Tunnel St+ and St- contained considerable amounts of road dust (mineral particles), Diesel was used as a reference for vehicle exhaust without mineral particles. The four samples (Tunnel St+, Tunnel St-, Wood and Diesel) were all collected as total suspended particulate matter (TSP).

### Electron microscopy

The particles were dispersed in distilled water, and sonicated for 30 min in a water bath. Two μl of the solution was placed on a carbon filmed 200 mesh copper grid, or a microgrid with openings of approximately 0.1 – 4 μm. To avoid interference with the amorphous carbon film, HRTEM, SAED and EELS was performed on primary particles suspended over holes in the microgrid. The particle morphology and geometric diameters were examined by a TEM (JEM-1010, Jeol, Japan), operated at 100 keV. Since carbon aggregates were often linked in networks covering large areas, exact numbers of aggregates were not possible to count. Semi-quantification of the relative amounts of carbon aggregates and mineral particles was, therefore, used to describe specimens containing only (+++), mostly (++), or little (+) of the respective particles (Table 1). Geometric diameters of approximately 300 primary carbon particles were measured for each sample, in 10–15 carbon aggregates chosen on a random basis (method described in Kocbach et al. (2005) [26]). The microstructure of primary carbon particles was investigated using a HRTEM (JEOL-2010F, Jeol, Japan) operated at 200 keV, and attached to a parallel-detection EELS spectrometer (GIF; Digital Micrograph 2.5). Electron energy loss spectra were recorded in TEM imaging mode with an energy dispersion of 0.2 eV per channel. Background subtraction of the spectra was performed using the Gatan EELS software EL/P 3.0. The heights of the π* and σ* peaks in the carbon K-edge were used to determine the π*/σ* ratios, in order to investigate the graphitic character of the primary particles [27]. The π*/σ* ratio was calculated for 8–13 spectra from primary particles in each sample and for the amorphous carbon film.

**Table 1:** Results from transmission electron microscopy. The semi quantitative amounts of carbon aggregates and mineral particles (see text) and mean values with standard deviations for the geometric diameters of primary particles.

Sample	Carbon aggregates	Mineral particles	Mean diameter (nm)
Tunnel St+	+	++	25 ± 7
Tunnel St-	++	+	24 ± 6
Wood	+++	-	31 ± 7 *
Diesel	+++	-	24 ± 7

* Significantly higher mean value

### Carbon analysis

The total carbon (TC) content of the samples was analysed by thermal optical transmittance, and divided into the organic (OC) and elemental (EC) carbon fractions. An instrument from Sunset Laboratories Inc (USA) was used for the analysis, according to the National Institute of Occupational Safety and Health (NIOSH) method 5040 [28]. The applied temperature steps during the first stage (pure He atmosphere) were 220°C (60 sec), 360°C (60 sec), 525°C (60 sec) and 850°C (90 sec), and during the second stage (98% He and 2% O_2_) 550°C (30 sec), 650°C (30 sec), 720°C (30 sec), 790°C (40 sec), 820°C (30 sec), 860°C (20 sec) and 890°C (40 sec). The quartz filters (1.5 cm^2^) used for the analysis were heated prior to particle application, to minimise the carbon background level of the filters. For each sample, particles were applied to 3–5 quartz filters which were weighed before and after particle application, to determine the particle mass. An organic carbon contamination during particle application was determined to be 0.28 ± 0.04 μg C/cm^2 ^by analysis of three filters. This was subtracted from the organic carbon levels.

### PAH analysis

A total of 18 PAHs were selected for analysis, including the US Environmental Protection Agency PAH 16, which are priority PAHs based on concerns that they cause or might cause cancer in animals and humans. Additionally, two PAHs commonly measured in ambient air samples, Benzo(e)pyrene and 1-Methylphenanthrene, were included. These may be carcinogenic but the evidence is limited or inadequate [29]. The PAH content in Tunnel St+, Tunnel St- and Wood was analysed by GC-MS at the National Institute for Public Health and the Environment (RIVM, Bilthoven, the Netherlands). An aliquot (1 ml) of internal standards (16 deuterated PAHs) and 50 ml dichloromethane/isohexane (1:1) was added, and the compounds were released by sonication. After centrifugation the extract was concentrated by evaporation to dryness and redissolved in 1 ml toluene. The extracts were then filtrated over a low-binding Durapore PVDF membrane (0.22 μm) with a final extract volume of 1 ml. One μl was injected (splitless mode) at 290°C on a 30 m, 0.25 mm WCOT DB-5MS column (film 0.1 μm). A column temperature programmed from 90 – 160 – 300°C was used, in a Fisons 8000 series gaschromatograph equipped with an Interscience Voyager mass-spectrometer with EI in SIR mode. Detection limits were approximately 0.1 – 0.7 ng/extract. The method has been tested to give maximum yield for PAHs.

### Statistics

All statistical analyses were performed in SPSS 12.0.1. Differences in mean primary particle diameters and carbon content were analysed using parametric tests (one way ANOVA, independent samples t-test), while differences in π*/σ* ratios were analysed using non-parametric tests (Kruskal-Wallis, Mann-Whitney). Probability levels less than 0.05 were considered statistically significant. For multiple testing, the *p*-values were adjusted according to the Sequential Bonferroni approach [30].

## Results

### Particle morphology (TEM)

**Figure 1:**
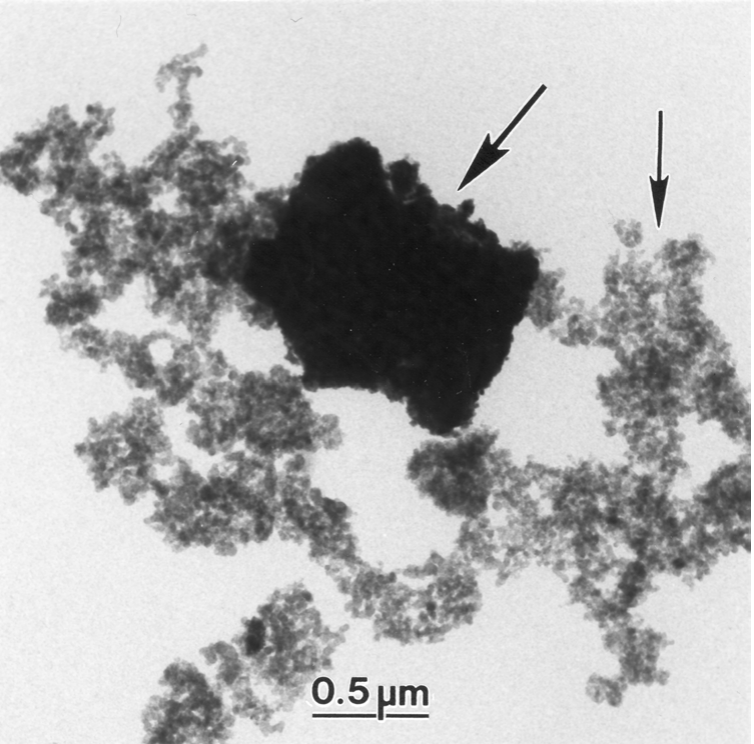
**Particle morphology**. TEM micrograph of a tunnel sample showing a mineral particle (large arrow) and carbon aggregates (small arrow).

The collected tunnel samples contained carbon aggregates, consisting of tens to thousands of primary carbon particles, and mineral particles of arbitrary shape (Figure 1). In contrast, Wood and Diesel contained only carbon aggregates. As could be expected, Tunnel St+ contained more mineral particles relative to the amount of carbon aggregates, as opposed to Tunnel St- (Table 1). Moreover, Tunnel St+ contained larger sized mineral particles (60% > 2 μm) as compared to Tunnel St- (20% > 2 μm). The size distributions of the geometric diameters of primary carbon particles were approximately normally distributed and monomodal for all samples (not shown). As seen in Table 1, the mean diameter for Wood was significantly larger than the mean values for the three vehicle exhaust samples (Tunnel St+, Tunnel St-, and Diesel). There were no significant differences between any of the vehicle exhaust samples.

### Microstructure of primary carbon particles

**Figure 2:**
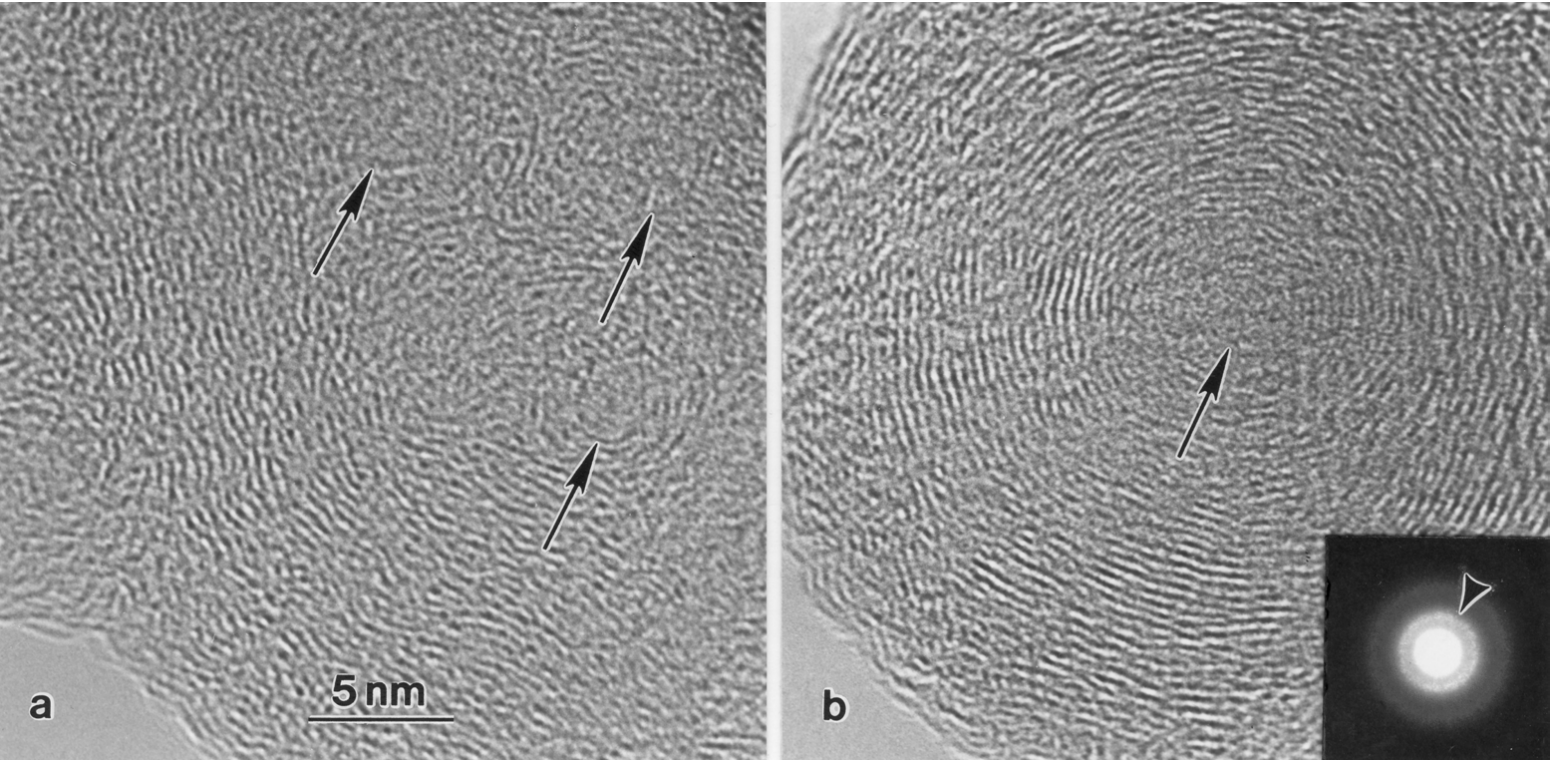
**Turbostratic microstructure of primary carbon particles**. TEM micrographs showing the turbostratic microstructures, consisting of concentric carbon layers surrounding a) several nuclei in vehicle exhaust (arrows), or b) a single nucleus in wood smoke (arrow). The inset shows a SAED pattern from a wood smoke particle. The arrowhead points at the ring corresponding to the 002 spacings in the turbostratic microstructure.

Using HRTEM, a turbostratic microstructure consisting of concentric carbon layers surrounding either several nuclei or a single nucleus, was observed for primary particles in samples from both vehicle exhaust and wood smoke. In the vehicle exhaust samples, primary particles with several nuclei were observed most frequently (Figure 2a), in contrast to the wood smoke sample were only particles with one nucleus were found (Figure 2b). The observed turbostratic microstructure was confirmed by the presence of a 002 ring in the SAED patterns (inset in Figure 2b). Calculation of the interlayer distances from the diffraction patterns gave values between 0.38 and 0.40 nm. No differences were, however, found between the samples.

**Figure 3:**
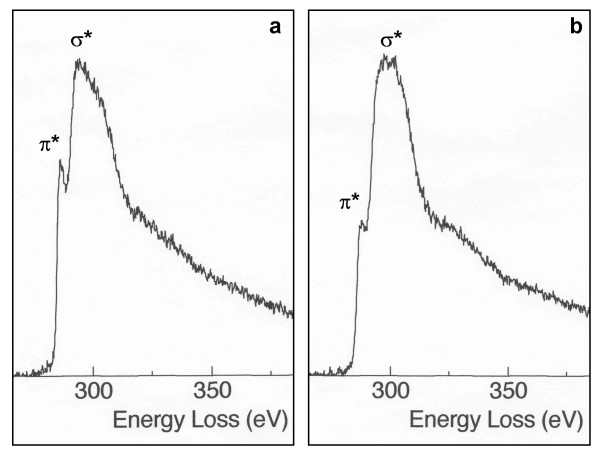
**EELS spectra**. The spectra show the π* and σ* peaks in the carbon K-edge for a) a primary carbon particle (wood smoke) and b) the amorphous carbon film.

To further investigate a possible difference in the graphitic character of primary particles from vehicle exhaust and wood smoke, the samples were analysed by EELS. The spectra from primary particles from both sources showed a higher ratio for the π* peak to the σ* peak than spectra from an amorphous carbon film (Figure 3a and 3b, respectively), indicating a graphitic structure [27]. The mean π*/σ* ratios for primary particles in all four samples were significantly higher (0.56 ± 0.05 – 0.60 ± 0.04) than for the carbon film (0.47 ± 0.05), but with no significant differences between the samples.

### Carbon analysis

Total carbon (TC) was determined to estimate the relative content of carbonaceous and mineral particles in the samples. As seen from Table 2, the mean TC mass percentages differed significantly between the samples, except for Wood and Diesel. The large difference in TC percentages for Tunnel St+ and St- reflects the difference in mineral particle content as observed by TEM. Accordingly, the high TC percentages for Wood and Diesel reflect that these samples contain only carbon aggregates.

**Table 2:** Results from chemical bulk analysis. The mean values with standard deviations for mass percentages of TC and OC, and OC/TC ratio, as well as the Total PAH and Adjusted PAH (see text).

Sample	TC (%)	OC/TC (%)	OC (%)	Total PAH (ng/mg)	Adjusted PAH (ng/mg)
Tunnel St+	14.3 ± 0.1	65.1 ± 3.1	9.3 ± 0.4	73	510
Tunnel St-	51.0 ± 3.8	47.9 ± 0.3*	24.4 ± 2.0	381	747
Wood	82.6 ± 5.8*	42.7 ± 4.4*	35.4 ± 5.1	9745	11797
Diesel^x^	80.0 ± 5.1*	20.4 ± 1.1	16.3 ± 1.2	67	84

^x^ The PAH sums for Diesel are based on data from the Certificate of Analysis (see text).

* Pairs of data where no significant differences were observed

In Table 2, the ratio of organic to total carbon (OC/TC ratio) is given rather than the EC/TC ratio, since the toxicity of carbonaceous particles seems to be more closely linked to the organic rather than the elemental fraction of the particles. The mean OC/TC ratios differed significantly between the samples, except for Tunnel St- and Wood. No source difference was found in OC/TC ratio, since the ratio for Wood was within the range of the three vehicle exhaust ratios. With respect to biological experiments, the mass percentage of organic carbon (Table 2), which differed significantly between all samples, is more relevant than the OC/TC ratio.

### PAH analysis

To compare the PAH content in the samples, the sum of the 18 measured PAHs (Total PAH) is given for each sample in ng/mg (Table 2). The Total PAH for Diesel is based on data from the Certificate of Analysis [31], and does not include measurements of Fluorene, Acenaphthene, Acenaphthylene and Naphthalene. As seen in Table 2, the Total PAH was considerably higher in Wood than in the vehicle exhaust samples. However, the TEM analyses and TC mass percentages showed that the tunnel samples contained significant amounts of mineral particles. To be able to compare the PAH content of the combustion particles in the tunnel samples, to that of Wood and Diesel (containing only combustion particles), the PAH content relative to the mass fraction of TC was calculated for all samples. This Adjusted PAH content (Table 2) was also considerably higher for Wood, compared to the tunnel samples, confirming that the observed source difference was not due to the mineral particle content.

**Figure 4:**
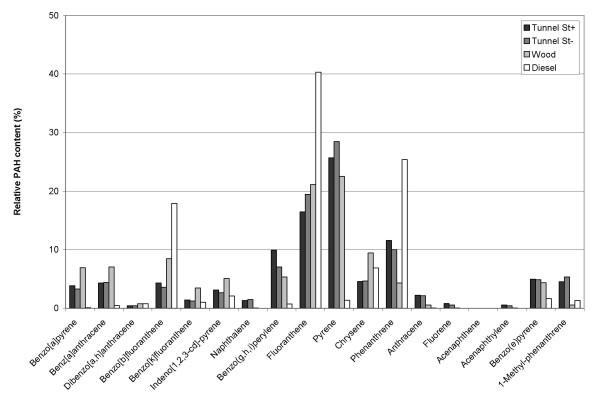
**PAH profiles**. The histogram shows the levels of single PAHs divided by Total PAH for the four samples (Tunnel St+, Tunnel St-, Wood and Diesel).

The PAH profiles for the four samples, presented as a histogram in Figure 4, show levels of the single PAHs divided by the Total PAH. The profiles were found to be quite similar for Tunnel St+, Tunnel St- and Wood with Pyrene and Fluoranthene as the dominating compounds, whereas the profile for Diesel differed considerably having a higher content of Fluoranthene, Phenanthrene and Benzo [b]fluoranthene.

## Discussion

Road traffic and residential wood combustion are the two principal sources of particulate air pollution in Norway. Differences in physical and chemical characteristics of combustion particles from these two sources may influence the severity of the health effects induced by inhalation of the particles. Since the samples analysed in the present study are also used to examine biological activity, the sampling sites and methods were chosen to provide large quantities. A vehicle exhaust sample without mineral particles (Diesel) was included in the study, since the biological effects of mineral particles may be higher than for carbonaceous particles in some biological systems [32-34].

Carbon aggregates were the only combustion particles observed in all samples, in agreement with previous observations in ambient air source samples [26]. Additionally, mineral particles most likely originating from road abrasion were observed in the two tunnel samples. The higher mineral particle content observed in Tunnel St+ compared to Tunnel St- was probably due to the use of studded tires during the sampling period. In agreement with our previous study of low volume collection of ambient air particles [26], the primary particle diameter in carbon aggregates from wood smoke was found to be significantly higher compared to vehicle exhaust. However, the diameters reported previously, were somewhat larger (27 ± 7 nm for vehicle exhaust and 38 ± 11 nm for wood smoke), probably due to differences in TEM specimen preparations. In our previous study, carbon extraction replicas coated with a 15 nm carbon film, were prepared directly from the collection filters.

The turbostratic microstructure of primary particles in samples from mixed vehicle exhaust and residential wood smoke has not been described in the literature. The microstructure observed in the vehicle exhaust samples, however, with several nuclei surrounded by concentric carbon layers, resembles the turbostratic microstructure described for carbon black [17] and diesel exhaust [19]. The microstructure of a particle may give information about the processes involved in particle growth, condensation and coagulation [19,35]. The present results suggest that growth of wood smoke particles is dominated by condensation (one nucleus), while growth of vehicle exhaust particles involves a combination of coagulation and condensation (several nuclei).

The interlayer distances calculated from the SAED patterns were not found to differ between primary particles from vehicle exhaust and wood smoke, probably since the diffraction patterns were broad and diffuse, making an accurate measurement of the diameters difficult. The calculated interlayer distances are, however, in reasonable agreement with those reported for diesel exhaust particles, which range from 0.36 nm [23,36] to 0.383 ± 0.025 nm [21]. A possible source difference in graphitic character was further investigated by EELS, as previously applied by Katrinak et al. (1992) [27] and Jaeger et al. (1999) [18], but no significant differences were found.

The low TC mass percentage observed for Tunnel St+ compared to Tunnel St-, is thought to be a consequence of the higher mineral particle content due to the use of studded tires. The OC/TC ratios measured by thermal optical transmittance, are reported to range from 8–85% for diesel vehicles [37-39] and 69–98% for gasoline vehicles [38]. The OC/TC ratios presently measured for Tunnel St+ and St- (Table 2) are lower than those reported for gasoline. This may indicate a higher contribution from diesel than gasoline vehicles, which is in line with the higher relative contribution from diesel (88%) compared to gasoline (12%) vehicles, to the particulate vehicle emission estimates for Norway [24]. Incidentally, traffic counts in the road tunnel showed lower counts for long vehicles (> 5.5 m, diesel vehicles) during the sampling of Tunnel St+ compared to Tunnel St- (Harald Granrud, Norwegian Public Roads Administration, personal communication). This may explain, in part, the difference in OC/TC ratio between the two tunnel samples. The wood smoke sample analysed, in the present study, has considerably lower OC/TC ratio (42.7%) than reported for residential wood smoke in the literature (80–98%) [38,40]. A possible explanation is that Wood was collected during high-temperature combustion and did not contain substances from the devolatilization of the wood, while the samples analysed in the literature were collected during the entire burning cycle. Moreover, this may also explain why no source difference was found in OC/TC ratio in the present study, whereas the ratios reported for wood smoke in the literature [38,40] are mostly in a higher range than for vehicle exhaust [37-39].

The PAH levels reported in the literature for source emissions are mostly given as ambient air concentrations (ng/m^3^) or emission factors (ng/km or ng/kg), making a comparison with other data difficult. However, a recent study reported fractional mass abundances (ng/mg) of PAH for a variety of vehicle exhaust source samples [41] with the highest PAH content in emissions from gasoline cars (3420 and 6900 ng/mg), and considerably lower values for diesel vehicles (273 and 444 ng/mg) and road dust (0.61–2.2 ng/mg). Comparison of these data with the present results (Adjusted PAH, Table 2) suggests that the tunnel samples, after adjusting for total carbon, can be considered to be source specific samples for mixed vehicle exhaust.

The PAH profiles for the tunnel samples correspond well with some reported road dust profiles [41,42]. In contrast, Wood differs from all profiles reported in the literature, with a lower content of Phenanthrene and Anthrachene [43,44] or Acenaphthylene and Naphthalene [45]. This may be due to sampling from combustion of different wood species and variable combustion conditions. Moreover, differences in sampling methods in the various studies may also influence the obtained profiles. Although some information about the carcinogenic and mutagenic potency of the analysed PAHs is available [16,29,42,46,47], their relative potencies are not identified. However, there is agreement in the literature that Benzo(a)pyrene is the most carcinogenic PAH, and that several have been found to be mutagenic [16,46]. Since the PAH profiles for Wood and the tunnel samples were found to be similar, the higher content of PAHs in the wood smoke particles indicates a higher mutagenic potential compared to vehicle exhaust. The actual contribution from wood combustion to ambient particle concentrations is likely to vary considerably with location and season, and the human exposure may be lower than the emission estimates for wood combustion of 65% [24]. However, based on ambient air measurements in Elverum, Norway, the contribution from residential wood combustion to fine particulate carbon has been estimated to be 65% [48], confirming that residential wood combustion is indeed a very important source to particulate air pollution in Norway.

The present results suggest that combustion particles from vehicle exhaust are characterised by a larger surface area to mass and a lower content of organic carbon and PAHs, as compared to wood smoke particles. Studies using particles such as polystyrene, carbon black and TiO_2_, *in vitro *and *in vivo*, have demonstrated that the particle toxicity increases with surface area [7-9,49,50]. Moreover, a high content of organic carbon and PAHs has been associated with increased inflammatory and oxidative potential [12-14]. Based on the literature it is, however, not possible to assess the relative influence of surface area and organic content on particle toxicity. Possible differences in the toxicity of combustion particles from vehicle exhaust and wood smoke could, therefore, not be determined based on the present results. In order to compare the toxicity of particles from the two sources, biological experiments need to be conducted.

Although the analysed wood sample is only representative for high temperature combustion, and the reference diesel sample for older cars, they both contain carbon aggregates only, and allow for comparison of combustion particles from the two sources. The four samples differ in particle composition (carbon aggregates/mineral particles), primary particle diameter, OC mass percentage and PAH content and profile, and are therefore considered suitable for assessing the influence of these characteristics on the biological potential of particles.

## Conclusion

The TEM analyses showed that combustion particles from vehicle exhaust and wood smoke differ in geometric primary particle diameter and turbostratic microstructure, whereas no significant differences were found in graphitic character using EELS and SAED. The bulk chemical analyses did not show any clear source difference in OC/TC ratio, whereas the PAH content was found to be much higher in wood smoke as compared to vehicle exhaust. The relative toxicity of combustion particles from the two sources could not be determined based on the present results and the available literature. A higher mutagenic and carcinogenic potential of the wood smoke particles seems likely, however, in the light of the similar PAH profile but larger PAH content compared to vehicle exhaust. With respect to biological experiments, the analysed samples seem more suitable for assessing the importance of physicochemical characteristics for biological responses, than for comparing the biological potential of combustion particles from the two sources.

## Abbreviations

ANOVA – analaysis of variance; EC – elemental carbon; EELS – electron energy loss spectroscopy; GC-MS – gas chromatography-mass spectrometry; HRTEM – high resolution electron microscopy; OC – organic carbon; PAH – polycyclic aromatic hydrocarbon; SAED – selected area electron diffraction; SRM – standard reference material; TC – total carbon; TEM – transmission electron microscopy.

## Competing interests

The author(s) declare that they have no competing interests.

## Authors' contributions

AK designed and coordinated the experimental work of this study, collected the samples, prepared all specimens for electron microscopy and carbon analyses, performed TEM and participated in carbon analyses. Furthermore, AK processed all data including tables and figures, carried out the statistical analyses, interpreted the results, wrote and prepared the manuscript. YJL performed the HRTEM, SAED and EELS. KEY performed the carbon analyses. FRC was responsible for the PAH analyses and participated in writing of the manuscript. PES participated in study design and writing of the manuscript. EN participated in study design, supervised the experimental work, participated in interpretation of the results, and is a co-writer of the manuscript. All authors read and approved the final manuscript.
